# Biopolymers and Their Application in Bioprinting Processes for Dental Tissue Engineering

**DOI:** 10.3390/pharmaceutics15082118

**Published:** 2023-08-10

**Authors:** Suhon Kim, Hanjun Hwangbo, SooJung Chae, Hyeongjin Lee

**Affiliations:** 1 Barun Plant Orthodontics and Dental Clinic, Seongnam 13312, Republic of Korea; suuhonkim@gmail.com; 2 Department of Precision Medicine, Sungkyunkwan University School of Medicine, Suwon 16419, Republic of Korea; hhwangbokorea@gmail.com (H.H.); sjchae65@gmail.com (S.C.); 3 Department of Biotechnology and Bioinformatics, Korea University, Sejong 30019, Republic of Korea

**Keywords:** polymers, 3D printing, dental tissues, tissue engineering

## Abstract

Dental tissues are composed of multiple tissues with complex organization, such as dentin, gingiva, periodontal ligament, and alveolar bone. These tissues have different mechanical and biological properties that are essential for their functions. Therefore, dental diseases and injuries pose significant challenges for restorative dentistry, as they require innovative strategies to regenerate damaged or missing dental tissues. Biomimetic bioconstructs that can effectively integrate with native tissues and restore their functionalities are desirable for dental tissue regeneration. However, fabricating such bioconstructs is challenging due to the diversity and complexity of dental tissues. This review provides a comprehensive overview of the recent developments in polymer-based tissue engineering and three-dimensional (3D) printing technologies for dental tissue regeneration. It also discusses the current state-of-the-art, focusing on key techniques, such as polymeric biomaterials and 3D printing with or without cells, used in tissue engineering for dental tissues. Moreover, the final section of this paper identifies the challenges and future directions of this promising research field.

## 1. Introduction

The field of tissue engineering has witnessed remarkable progress in recent years, offering new avenues for regenerative medicine [1,2]. In dentistry, tissue engineering holds immense potential for the restoration and regeneration of dental tissues, revolutionizing conventional approaches to dental treatments [3,4,5,6]. Dental tissue engineering aims to overcome the limitations associated with traditional restorative techniques by promoting the growth and regeneration of dental tissues, including dentin, cementum, and periodontal ligaments cultured on various scaffolds [7].

The regeneration of dental tissues poses significant challenges because of their complex hierarchical structures, functional requirements, and innate regenerative limitations [8]. Advancements in biomaterials, cell-based therapies, and tissue engineering strategies have provided innovative solutions to these challenges [9,10,11]. Dental tissue engineering endeavors to replicate the natural regenerative processes that occur during tooth development and repair. It involves the use of biocompatible scaffolds, growth factors, and stem cells to create an optimal microenvironment that supports cell adhesion, proliferation, and differentiation [12,13]. Scaffolds act as three-dimensional (3D) frameworks, providing structural support and guiding the growth and organization of cultured cells. By mimicking the composition of the extracellular matrix (ECM) and architecture of dental tissues, these scaffolds facilitate the formation of functional tissue constructs [14].

A variety of polymer-based biomaterials have recently been explored for scaffold fabrication in dental tissue engineering, including the biodegradable polymers poly(lactic-co-glycolic acid) (PLGA) [15,16], polycaprolactone (PCL) [17], and collagen-based materials [18]. These biomaterials have demonstrated favorable properties, including biocompatibility, tunable degradation rates, and mechanical integrity. Furthermore, the incorporation of bioactive molecules, including growth factors, peptides, and nanomaterials, into these scaffolds has shown promising results in enhancing cell behavior, tissue regeneration, and mineralization [19,20,21].

In recent years, the emergence of 3D bioprinting has revolutionized the fabrication of complex scaffolds for dental tissue engineering [22]. This additive manufacturing technique enables precise control over scaffold architecture, porosity, and spatial distribution of cells and bioactive factors. By employing computer-aided design software and a layer-by-layer deposition of biomaterials, 3D bioprinting allows for the creation of patient-specific scaffolds with tailored mechanical and biological properties [23]. Furthermore, the incorporation of multiple cell types within these constructs has shown potential for the regeneration of multi-tissue interfaces, including the dentin-pulp or periodontal ligament-bone complex [24].

The future of dental tissue engineering holds great promise. Advancements in biomaterials, scaffold design, stem cell research, and biofabrication techniques have enabled the development of efficient and effective strategies for dental tissue regeneration. The integration of innovative technologies, including gene editing, tissue-on-a-chip systems, and in vitro organogenesis, is likely to further augment the regenerative capabilities of dental tissue engineering approaches.

In this review, we aim to explore the current state-of-the-art in dental tissue engineering, particularly highlighting polymeric biomaterials employed for the regeneration of dental tissues and the emerging role of 3D bioprinting in polymeric scaffold fabrication. By elucidating the current progress in, and prospects of, dental tissue engineering, this review aims to inspire further research and foster the development of novel strategies for successful dental tissue regeneration.

## 2. Conformation of Dental Tissues and Polymeric Scaffolds

**Figure 1:**
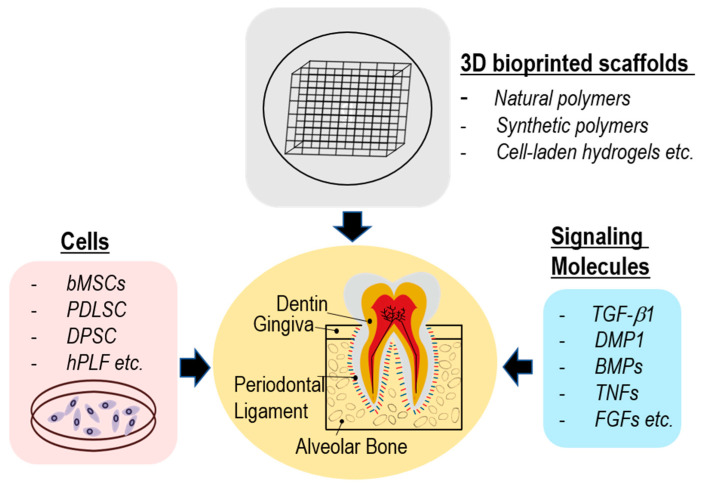
A schematic representation delineating the tissue engineering approach for the regeneration of periodontal tissues, which were obtained using 3D bioprinted scaffolds, diverse stem cells, and signaling molecules.

There are three main types of dental tissues: enamel, dentin, and cementum. Enamel, known as the crown, is the outermost layer and covers the visible part of the tooth. It is primarily composed of hydroxyapatite crystals, which provide its hardness and strength; it does not contain living cells and cannot regenerate once it is formed. The main functions of the enamel are to protect the underlying dentin and provide a smooth surface for chewing and speaking. Dentin lies beneath the enamel and cementum, makes up the majority of the tooth structure, and is a mineralized tissue that contains hydroxyapatite crystals, collagen fibers, and fluid-filled tubules (Figure 1). Dentin is not as hard as enamel but can provide support and strength to the tooth. Unlike enamel, dentin contains living cells called odontoblasts, which are found at the interface between the dentin and the pulp cavity. Odontoblasts play a role in dentin formation and secrete new dentin in response to various stimuli, including tooth decay or trauma [25,26]. Various signaling molecules, such as transforming growth factor beta 1 (TGF-β1), dentin matrix acidic phosphoprotein 1 (DMP1), bone morphogenetic proteins (BMP), tumor necrosis factor alpha (TNFα), and fibroblast growth factor (FGF), can modulate different signaling pathways that regulate cellular activities, such as cell proliferation, migration, and differentiation [27,28,29]. For example, a previous study reported that DMP-1 and TNFα are critical components for mineralization and odontogenesis [30], while TGF-β1 can stimulate dentinogenesis through Wnt/β-catenin signaling [31]. Furthermore, BMP can enhance the osteogenic activities of cells [32], while FGF can induce pro-angiogenic effects [33].

Cementum covers the root of the tooth and helps anchor the tooth to the surrounding alveolar bone through the periodontal ligament. It is a mineralized connective tissue containing collagen fibers, hydroxyapatite crystals, and living cells known as cementocytes [34]. Cementum is not as hard as dentin or enamel but plays a crucial role in tooth stability and supports the periodontal ligament fibers. Similar to dentin, cementum has a limited ability to repair itself by forming new layers in response to external factors.

Among these dental tissues, dentin tissue has been investigated using scaffold-based tissue regeneration approaches with synthetic polymers, including poly(lactic acid) (PLA), poly(glycolic acid), PCL, and natural polymers (collagen, gelatin, chitosan, alginate, and hyaluronic acid). Furthermore, a combination of synthetic polymer and natural biopolymers or hydroxyapatite nanoparticles can enhance the mechanical strength and bioactivity of the scaffold, promoting dentin regeneration [35,36]. Specifically, dentin tissue engineering often focuses on utilizing polymeric scaffolds to promote the proliferation and differentiation of odontoblast-like cells. Polymeric scaffolds facilitate the regeneration of dentin-like tissues by providing a suitable environment for odontoblast-like cells.

Generally, polymeric scaffolds used in dentin tissue engineering are designed to have a porous structure, allowing the infiltration of cells and exchange of nutrients and waste products [37]. In addition to providing a physical scaffold, polymeric materials can be functionalized with bioactive molecules, growth factors, or signaling molecules to enhance cell attachment, proliferation, and differentiation [38,39]. These bioactive cues can mimic natural signaling pathways involved in dentin formation and guide the development of new tissues. As shown in Figure 1, by combining polymeric scaffolds with appropriate cells (dental pulp stem cells (DPSCs), stem cells from exfoliated deciduous teeth, periodontal ligament stem cells (PDLSCs), and dental follicle progenitor cells) and signaling molecules (TGF-β, BMPs, insulin-like growth factor, and platelet-derived growth factor), several researchers have investigated the feasibility and effectiveness of regenerating dentin-like tissue in vitro and potentially developed new strategies for dental tissue repair and regeneration.

## 3. Bioprinting for Dental Tissue Engineering

Advances in tissue engineering have led to the emergence of 3D bioprinting as a promising technique for fabricating complex dental structures [40]. Three-dimensional bioprinting enables the precise spatial organization of cells, biomaterials, and growth factors, allowing the creation of patient-specific and functional dental tissues. In this section, we explore various 3D bioprinting technologies used in dental tissue engineering.

**Figure 2:**
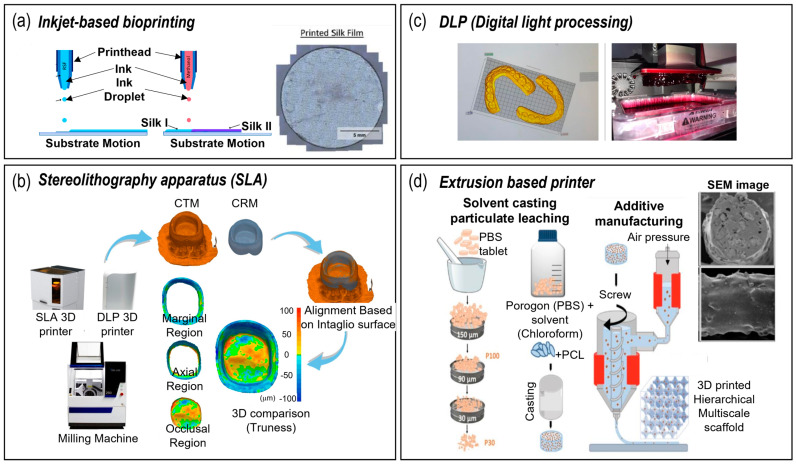
3D fabrication systems for dental tissue engineering. (**a**) Inkjet-based bioprinting [44]; (**b**) sterolithography [45]; (**c**) digital light processing [46]; and (**d**) extrusion-based fabrication method [47].

Inkjet-based bioprinting is a non-contact printing technique that utilizes thermal, piezoelectric, or microvalve processes to dispense droplets of dilute solutions (Figure 2a). It operates similarly to traditional inkjet printing, but uses bioinks containing cells and biomaterials [41]. This technology allows for a high spatial resolution, ranging from 50 to 300 μm [42], but the presence of cell aggregation within the bioink can affect droplet formation and trajectory, leading to a decrease in print quality [43]. Rider et al. utilized a reactive inkjet printing method to obtain high-resolution nanosized hydroxyapatite-incorporated silk fibroin membranes [44]. However, the use of low-concentration solutions can limit the construction of 3D structures for dental tissue engineering.

Laser-assisted bioprinting utilizes the power of laser beams to achieve the meticulous deposition of bioink substances onto a substrate. Stereolithography apparatus (SLA) is an example of laser-assisted 3D printing. Son’s team (2021) used SLA 3D printing to fabricate interim crowns for dental implantation (Figure 2b) [45]. Through localized pressure generated by the laser, the bio-ink was propelled in the form of droplets onto a designated target area. This cutting-edge technology offers the ability to achieve high-resolution printing, surpassing the threshold of 20 µm [48].

Such precision allows for the accurate placement of cells and biomaterials, thereby promoting intricate biological constructs. To ensure a successful process, the precursor material should be a hydrogel with a viscosity within a moderate range [49]. Furthermore, this method has demonstrated the potential to enable precise multicell positioning and facilitate intricate cellular arrangements [50]. However, the complexity involved in selecting the optimal printing conditions, such as gelation time and laser fluence, poses challenges, potentially resulting in a compromised cell viability. The requirement for expensive apparatus further complicates the widespread adoption of this technique [51].

Operating on a principle such as the SLA, the direct light processing (DLP) fabrication method utilizes localized light to solidify a photocross-linkable liquid to obtain 3D structures. DLP fabrication methods are regularly used to convert 3D models into 3D structures for dental implants (Figure 2c). Furthermore, DLP can polymerize each layer of resin much more rapidly compared to SLA, which is a preferable process [46].

Extrusion bioprinting is driven by a piston, screw, or pneumatic pressure mechanisms such that highly viscous bioinks can be printed through micronozzles [52]. Conventional extrusion-based printing systems are regularly used to fabricate scaffolds for guided dental regeneration. For instance, Figure 2d illustrates the incorporation of salt microparticles in biopolymers (PCL), as recently investigated in 2023 [47]. The subsequent leaching of salt particles after fabrication allows for the formation of micro/macroporous scaffolds for dental tissue engineering. This technique is versatile and compatible with a wide range of biomaterials including hydrogels and biopolymers. In particular, for cell-laden structures, this technique enables the printing of very high cell densities with a fast rate of fabrication [53,54]. However, there are potential cell apoptotic effects induced during and after printing owing to the pressure drop associated with extrusion through a micronozzle [51,55].

Bioprinting can be used in the regeneration of intricate dental structures with the precise spatial organization of diverse tissues. This facilitates the regeneration of vascularized pulp-like tissue and the formation of mineralized tissue within stem cell constructs by employing DPSCs and stem cells from the apical papilla [56]. Bioprinting also plays a role in the regeneration of integrated cementum on the surface of the roots of a human tooth. This was achieved by utilizing growth-factor-releasing scaffolds containing PDLSCs, which were incorporated into 3D-printed PCL scaffolds. The research team (2017) has further enhanced the scaffold with PLGA microspheres encapsulating connective tissue growth factor, BMP-2, or BMP-7 [57].

The application of 3D bioprinting in dental tissue engineering offers great potential for personalized dental treatment and the rapid prototyping of dental structures. Although some challenges remain, including the scalability of the technique, optimization of bioink formulations, and long-term functionality of printed tissues, 3D bioprinting is a rapidly evolving field that holds extensive promise.

## 4. Polymeric Materials and Their Printed Scaffolds for Dental Tissue Engineering

The choice of scaffold material is critical for dental tissue engineering, as it affects the cellular activities and the tissue regeneration. Both biological and mechanical properties are important factors for dental tissue regeneration. The biological properties influence the bioactivities of the cells, while the mechanical properties ensure structural stability under dynamic loadings. Generally, natural polymers can provide suitable biochemical cues, thus enhancing the bioactivities of cells, whereas synthetic polymers can provide adequate mechanical properties. This section of the review discusses various studies that use natural, synthetic, and hybrid polymers for dental tissue engineering.

### 4.1. Natural Polymers

Natural scaffold materials derived from biological sources offer several advantages, including biocompatibility, biodegradability, and the ability to mimic the composition and structure of native ECM. Commonly used natural scaffold materials in dental tissue engineering include collagen, gelatin, chitosan, hyaluronic acid, fibrin, and decellularized ECM (dECM).

Collagen, derived from several sources, including bovine, porcine, or human tissues, is a versatile scaffold material that supports cell adhesion and promotes the regeneration of dental tissues, including dentin, periodontal ligament, and gingiva. This is due to its anatomically similar structure and chemical properties to the predominant structural proteins existing in the ECM of dental tissues [58,59].

Although collagen has high tensile strength and can be used in fibrous forms and load-bearing applications, it lacks sufficient mechanical strength for pulp regeneration [60]. Crosslinking collagen with glutaraldehyde or genipin enhances its mechanical properties. The combination of a collagen scaffold, DPSCs, and DMP1 improved the formation of ECM in pulp tissue [61], while Pandya et al. incorporated erythropoietin (EPO), a glycoprotein hormone that stimulates red blood cell production, into collagen scaffolds in 2021 [62]. They compared this with a commercially available BioOss inorganic bovine bone xenograft to investigate its potential for alveolar ridge augmentation. The incorporation of EPO into the collagen scaffold resulted in a two-fold increase in blood vessel formation and improvements in the deposition of ECM. Recently, Chang’s research team (2023) investigated a novel injectable cell-laden hydrogel consisting of collagen and riboflavin for the treatment of periodontal defects. They implemented a dental light-emitting diode crosslinking method to reinforce the injected hydrogel [63].

Gelatin, a derivative of collagen, possesses a porous structure that allows for cellular infiltration, making it suitable for regenerating dentin, periodontal tissues, and oral mucosa. Gelatin has been extensively used in both preclinical and clinical settings [64,65]. Gelatin offers distinct advantages over collagen, including reduced immune responses, more controllable physical properties, and a lower risk of unpredictable pathogen transmission. Importantly, gelatin exhibits favorable engineering properties, including ease of fabrication and the ability to control its mechanical properties during crosslinking [66,67]. Consequently, gelatin materials are increasingly considered in the application of advanced tissue engineering, particularly for manufacturing highly porous scaffolds that facilitate tissue repair and regeneration [68].

Alginate, a polysaccharide produced by a wide variety of brown seaweeds, is an effective cell carrier, scaffold, and delivery system for growth factors and bioactive materials used in regenerative endodontics. For example, Dobie et al. (2002) reported that TGF1 can upregulate the matrix secretion of dentin-pulp ECM, while the alginate polymeric matrix can serve as a delivery platform for various growth factors and bioactive components [69]. Zhang et al. (2020) fabricated alginate/laponite hydrogel microspheres that encapsulated DPSCs and vascular endothelial growth factor (VEGF) to develop injectable cell-laden microspheres for endodontic regeneration. The injection of these biomicrospheres induced vascularization after the subcutaneous implantation of tooth slices in nude mice. Additionally, the upregulation of odontogenic-related genes was observed [70].

Hyaluronic acid, a glycosaminoglycan found in the ECM presents hydration and lubrication properties. It has been widely used in scaffolds for cell adhesion and has shown promising results for regenerating periodontal tissues and oral mucosa. Park et al. (2003) has demonstrated that hyaluronic acid hydrogel modified with arginine–glycine–aspartic acid (RGD) has exhibited significant potential in promoting cell attachment and proliferation [71]. This highlights the favorable effects of RGD-modified hyaluronic acid hydrogels on cellular processes. Furthermore, the injectable nature of hyaluronic acid hydrogel allows it to penetrate narrow canals, making it highly suitable for applications in endodontics and pulp regeneration.

Fibrin, a structural component of blood clots, serves as a 3D framework that supports cell attachment, migration, and tissue formation. Compared to other natural polymers such as collagen, fibrin exhibits superior properties in terms of cell adhesion, biocompatibility, and immune response. However, it also possesses disadvantages, including high shrinkage rates, rapid degradation, and low mechanical strength [72]. To address these limitations, composite scaffolds comprising fibrin and biocompatible reinforcements, including hyaluronic acid, calcium phosphate, and polyurethane, significantly enhanced the mechanical properties of fibrin [73].

The use of fibrin as a biomaterial has several advantages. First, fibrinogen within its structure undergoes TGF-β transformation, leading to collagen formation [74]. Fibrin also provides a suitable environment for angiogenesis, exhibits the potential to control the release of proangiogenic growth factors [75], and offers injectability and the capacity to construct 3D structures. Because of these advantages, Zhang et al. (2020) utilized fibrin-based hydrogels as a delivery system for extracellular vesicles extracted from mesenchymal stromal cells to investigate the angiogenic effects in dental pulp regeneration [76] and observed favorable ECM deposition and a high angiogenic response.

dECM scaffolds are prepared by removing cellular components from natural tissues while preserving the structure and composition of the ECM. These scaffolds provide a biomimetic environment for tissue regeneration, and dental tissue-specific dECM scaffolds have been used to guide the regeneration of dental tissues. They provide structural support, promote cellular activity, and guide the regeneration of specific dental tissues, contributing to advancements in dental regenerative therapies. Various natural polymers have been used in dental tissue engineering, as summarized in Table 1.

### 4.2. Synthetic Polymers

Synthetic polymers, including PLGA, PLA, and PCL, are widely used for dental tissue engineering. These polymers exhibit tailorable mechanical properties, degradation rates, and biocompatibility. They can be fabricated in various forms, including films, fibers, and porous scaffolds, using multiple techniques, including electrospinning, solvent casting, and 3D printing. Synthetic polymer scaffolds have been utilized for the regeneration of several dental tissues, including dentin, periodontal ligament, and alveolar bone.

**Figure 3:**
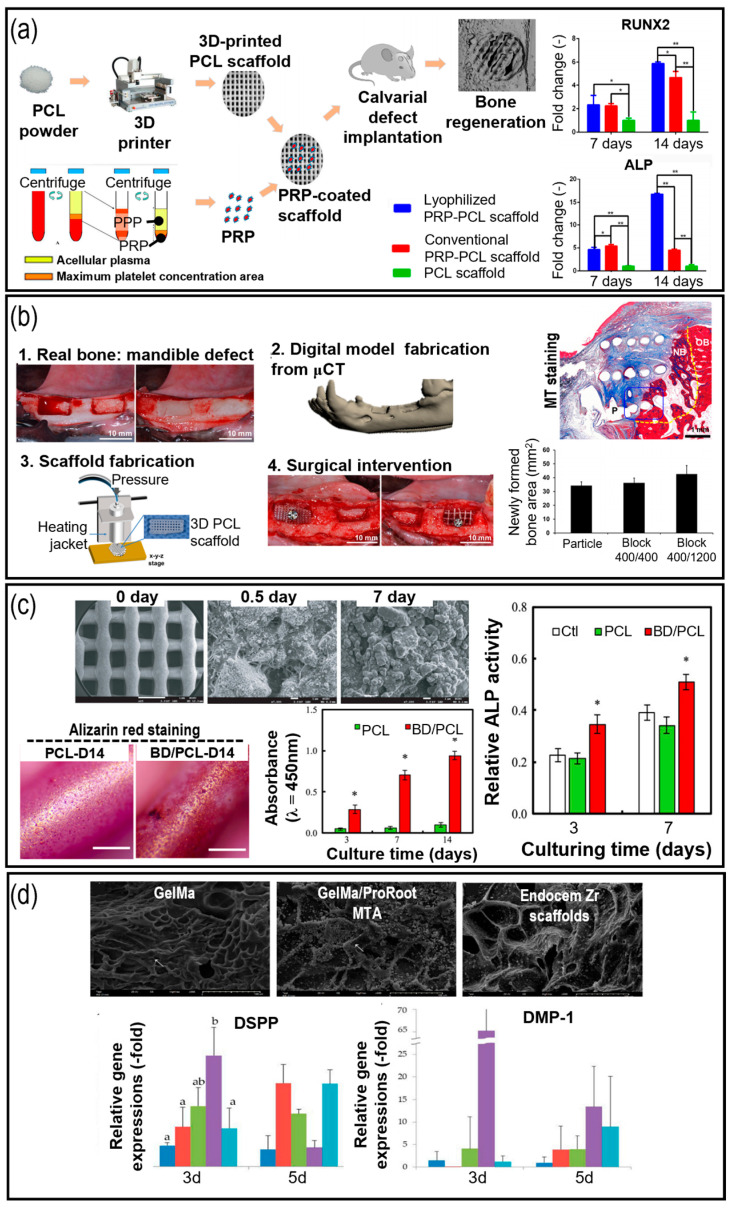
Application of polymeric materials used for dental tissue engineering. (**a**) Effects of platelet-rich plasma (PRP)-coated polycaprolactone (PCL) scaffold in osteogenesis of human dental stem cells (* *p* < 0.05 and ** *p* < 0.005) [93]; (**b**) implantation of β-TCP/PCL scaffold to alveolar defect in rats and Masson’s Trichrome (MT) staining results [95]; (**c**) SEM and ARS images of dentine incorporated PCL scaffold, cellular proliferation and alkaline phosphatase (ALP) activities of human dental pulp stem cells [96] (* *p* < 0.05); (**d**) incorporation of mineral trioxide aggregate to photo-crosslinkable methacrylated gelatin scaffolds (a, b, and ab, represents statistically significant differences (*p* < 0.05)) [97].

In 2021, alveolar bone regeneration was investigated through the incorporation of β-tricalcium phosphate (β-TCP) and platelet-rich plasma (PRP) into a PCL scaffold. Subsequently, bone marrow stem cells (bMSCs) were seeded onto a scaffold and implanted into mandibular bony defects in miniature pigs [84]. The incorporation of bMSCs and PRP into the PCL-TCP scaffold significantly increased the bone–implant contact ratio, the height of newly formed bone, and new bone formation, compared with the conventional PCL-TCP scaffold. Similarly, Li et al. demonstrated that the incorporation of PRP into PCL scaffolds can induce substantial osteogenic activity in DPSCs, in an experiment conducted in 2017 (Figure 3a) [93]. Tatullo et al. (2019) evaluated the osteo/odontogenic properties of a PLA scaffold modified with dicalcium phosphate dihydrate and/or hydraulic calcium silicate [85]. Human periapical cyst-derived mesenchymal stem cells (MSCs) cultured on the scaffold exhibited a 2.5-fold upregulation of DMP-1 gene expression compared to those cultured on pristine scaffold. However, the authors noted that the acidic degradation of PLA could hinder regeneration efficacy. Park et al. (2017) utilized an extrusion-based 3D printing system to fabricate PCL/β-TCP scaffolds [94]. Owing to the osteoinductive properties of β-TCP, significantly superior osteogenic properties were observed in MSCs lines than in pristine PCL. In 2018, implantation of the scaffold into alveolar defects in rats resulted in the improved formation of new bone (Figure 3b) [95].

Through the utilization of highly porous polymeric scaffolds, researchers have successfully regenerated dental tissues, including dentin and pulp tissues, in preclinical trials [98,99]. Additionally, the incorporation of drug-loaded polymeric scaffolds holds great promise in controlling the rates of release of gene vectors, proteins, and growth factors, and in creating spatiotemporal microenvironments that support tissue growth and regulate cell activities, including differentiation, proliferation, and migration [100]. For instance, in 2018, biodentine (an FDA-approved drug used for dentin repair) was incorporated with PCL to develop bioactive scaffolds for dental/bone regeneration [96]. The incorporation of biodentine significantly improves the cellular proliferation and osteogenic activities of human dental pulp cells (Figure 3c). However, a key challenge in dental tissue engineering is the development of strategic approaches to optimize tissue formation using complex micron-scale geometries and biodegradable polymeric materials [101,102]. Thus, there is significant demand for customized or flexible methodologies that employ biopolymers to address complex geometries or non-standardized and unpredictable defects in the field of periodontal tissue engineering.

Synthetic hydrogels, including poly(ethylene glycol) (PEG) and poly(vinyl alcohol), consist of hydrophilic polymers that form 3D networks that can retain significant amounts of water and undergo mild biodegradation. These hydrogels create a hydrated environment similar to the native tissues, enabling cell encapsulation, nutrient diffusion, and growth factor distribution. Through chemical modifications, synthetic hydrogels can incorporate bioactive signals to enhance cell adhesion, proliferation, and differentiation. In dental tissue engineering, these hydrogels have found applications in regeneration of dental pulp and periodontal tissue engineering. Notably, RGD peptide-modified PEG hydrogels have demonstrated elevated cell adhesion and proliferation, while modifications in the molecular weight and photocross-linking of PEG have improved its mechanical properties without detrimental effects on encapsulated cells [103,104,105].

One noteworthy example involves fibrin-loaded PEG hydrogels, which exhibit significant potential as scaffolds for the growth and proliferation of DPSCs and PDLSCs. These hydrogels combined the mechanical support and angiogenic properties of PEG with the benefits of fibrin hydrogels [105]. The multiple applications of synthetic polymers in dental tissue engineering are summarized in Table 1.

### 4.3. Polymer-Based Hybrid or Composite Materials

Composite scaffolds combine the advantages of multiple materials to achieve synergistic effects. In dental tissue engineering, composite scaffolds often comprise a combination of natural and synthetic materials. For example, a composite scaffold can be formed by incorporating natural polymers, including collagen or chitosan, in synthetic polymers, including PLGA or PCL. These hybrid scaffolds offer improved mechanical properties, enhanced biocompatibility, and controlled degradation. Composite scaffolds have been utilized for the regeneration of several dental tissues, including enamel, dentin, periodontal ligaments, and bone. Ducret et al. (2019) incorporated chitosan into a fibrin polymeric matrix to prevent the growth of endodontic bacteria and promote the regeneration of dental pulp tissue [86]. The authors stated that chitosan-incorporated fibrin polymeric hydrogels could be injected into the endodontic space for antibacterial effects. Furthermore, chitosan–gelatin composite hydrogels have been investigated for alveolar bone regeneration. The implantation of bone MSCs seeded with chitosan–gelatin scaffolds into tooth sockets in rats resulted in significant improvements in new bone formation and neovascularization [91]. Yu et al. investigated the effects of 3D-printed alginate/gelatin hybrid scaffolds using human dental pulp cells. Owing to the improved printability of the hybrid bioink, complex 3D geometries were successfully fabricated, while the cellular proliferation and osteo/odontogenic activities of human dental pulp cells were elevated.

Bioactive ceramics, including hydroxyapatite, tricalcium phosphate, and bioglass have been widely used in endodontic applications because of their similarity to the mineral components of natural dental tissues and their high bioactivity [106]. Hybrid scaffolds are created by combining bioactive ceramics with synthetic polymers or natural scaffolds. The combination of bioactive ceramics with polymers enhances the mechanical properties and provides a favorable environment for cell attachment, proliferation, and mineralization [107,108]. Bioactive ceramic-based scaffolds have been employed for the regeneration of dental tissues, including enamel, dentin, and alveolar bone. In 2012, improved characteristics were demonstrated in an alginate scaffold incorporated with a nano-bioglass ceramic [109]. The modified scaffold exhibited enhanced attachment, growth, and alkaline phosphatase (ALP) activity in hPDLF. For example, the incorporation of calcium silicate and calcium sulfate into PCL scaffolds greatly improves calcium deposition in human dental pulp cells, as indicated by the more intense Alizarin Red S (ARS) expression, as recently demonstrated in 2022 [110]. Similarly, Choi et al. (2022) incorporated calcium silicate cement into photocross-linkable methacrylated gelatin (GelMA) to investigate the cellular activity of human DPSCs [97]. As a result, Dentin Sialophosphoprotein (DSPP) and DMP-1 genes were significantly upregulated, indicating the efficient odontogenic activity of the cells (Figure 3d). Nejad et al. (2012) fabricated a 3D PCL/calcium sodium phosphoslicate Bioglass (BG) composite and PCL/hydroxyapatite (HA) scaffold to investigate dentin and pulp tissues [111]. The incorporation of BG greatly upregulated osteo/odontogenic-related genes, including DSPP, osteocalcin (OCN), and DMP-1, compared to PCL/HA, indicating the significant potential of BG in alveolar bone regeneration.

Nanocomposite scaffolds incorporate nanoscale materials, including nanoparticles or nanofibers, into a scaffold matrix. These nanomaterials can be either natural (e.g., nanocellulose) or synthetic (e.g., carbon nanotubes). The integration of nanomaterials imparts unique properties to scaffolds, including enhanced mechanical strength, improved bioactivity, and controlled drug release. Nanocomposite scaffolds have shown promise in dental tissue engineering for applications in dentin regeneration, enamel remineralization, and drug delivery systems. Jiang et al. (2015) demonstrated the incorporation of electrospun aligned PCL-PEG nanofibers into a porous chitosan scaffold to evaluate the regenerative efficacy of periodontal ligaments. Two months after implantation into the periodontal defect in rats, the significant formation of aligned periodontal ligaments was observed, owing to the appropriate contact guidance for the cells [92]. In addition to these examples, various combinations of polymeric composites have been reported for dental tissue engineering, as summarized in Table 1.

## 5. Bioprinting Using Cell-Laden Hydrogel Bioinks in Dental Tissue Engineering

Multiple cell types, including DPSCs, odontoblasts, and periodontal ligament cells, can be bioprinted in specific arrangements to create tooth-like structures. In addition, by providing a supportive hydrogel and appropriate bioactive cues, this process can promote the differentiation and maturation of these cells, ultimately leading to the regeneration of functional tooth.

In particular, bioprinting allows for the precise fabrication of cell-laden constructs using hydrogel bioinks with a controlled internal architecture and complex geometries. Recently, cell constructs have been used for dental tissue engineering to mimic the natural ECM of dental tissues, providing structural support and guiding cellular behavior. The selection of polymeric hydrogels and their composition can influence the mechanical properties, rates of degradation, and bioactivity of cell constructs, thus affecting the outcomes of tissue regeneration.

**Figure 4:**
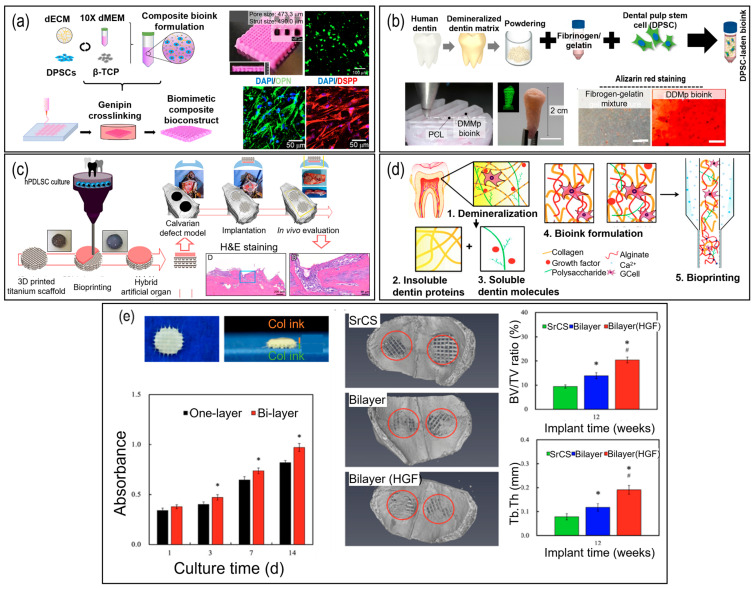
The application of cell-printing and cell-laden structures for dental tissue regeneration. (**a**) Application of bone decellularized extracellular matrix and β-tricalcium phosphate (β-TCP) for accelerated osteo/odontogenic differentiation of human dental pulp stem cells [112]; (**b**) incorporation of demineralized dentin matrix particles to fibronogen/gelatin/hyaluronic acid/glycerol to evoke efficient odontogenic differentiation [113]; (**c**) mechanically enhanced titanium/collagen hybrid structures for dental implants [115]; (**d**) incorporation of nano-sized demineralized human dentin matrix particles into alginate hydrogel for enhanced dental repair [116]; (**e**) top- and side-view photographic images of the Col bio-ink/SrCS bi-layer structure, the proliferation of encapsulated human gingiva fibroblasts (hGF), representative µCT images of osteoporotic rabbits’ cranial bone defect model after being implanted for 12 weeks, and µCT-quantified histograms of bone volume/total volume (BV/TV) and trabecular thickness (Tb.Th) (* indicates a significant difference (*p* < 0.05) compared to SrCS and # indicates a significant difference (*p* < 0.05) compared to bi-layer) [117].

To evaluate these effects, numerous studies have demonstrated the formulation of a dECM bioink (Figure 4a–e) [112,113,114,115,116,117]. Kim et al. (2022) investigated the odontogenic activity of a bone-derived dECM, (Figure 4a) [112]. The composition of bone-dECM was similar to that of a dentin-derived dECM. Significantly higher levels of expressions of osteopontin (*OPN*) and DSPP were observed in cells encapsulated in bone-dECM than in those encapsulated in collagen-based bioink. In 2021, human dentin-derived dECM cell constructs can significantly improve osteogenic activity (Figure 4b), as indicated by the more intense ARS staining of DPSCs cultured on fibrinogen–gelatin bioconstructs with demineralized dentin matrix particles [113]. Moreover, Buyuksungur et al. (2021) fabricated hybrid structures composed of synthetic (PCL) and natural polymer (GelMA) [114]. They printed PCL and cell-laden GelMA in alternating patterns, resulting in significant mechanical enhancements, suggesting the potential application in alveolar bone tissue regeneration. To further improve the mechanical properties, Lee et al. (2021) bioprinted hPDLSCs with collagen bioink onto 3D-printed titanium scaffolds (Figure 4c) [115]. The H&E staining showed that the hPDLSCs were well-proliferated in the calvarial bone defects after 6 weeks of implantation.

Another type of periodontal tissue, PDL, has also been studied using bioprinted structures. The bioprinting of PDL tissue using a GelMA hydrogel and PDLCs investigated the printability of different concentrations of the GelMA hydrogel as a bioink for constructing cell-laden structures. Various printing parameters, including photoinitiator concentration, UV exposure, pressure, and dispensing needle diameter, were evaluated to determine their influence on the viability of encapsulated periodontal ligament cells (2019). This research identified the most suitable printing conditions. By optimizing these printing parameters, the researchers were able to fabricate cell-laden constructs with a high printing resolution, dimensional stability, and favorable cell viability for periodontal ligament cells [118].

In recent years, dental tissue engineers have explored the use of cell-laden constructs fabricated using bioprinting techniques, which can function as carriers for controlled drug release. Targeted and sustained drug delivery can be achieved by incorporating bioactive molecules, growth factors, or antimicrobial agents into polymeric scaffolds. This has the potential to promote tissue regeneration, reduce inflammation, prevent infection, and achieve additional specific therapeutic objectives. 

The efficacy of drugs and growth factors in promoting the proliferation and differentiation of various dental stem cells has been extensively demonstrated. For example, Liu et al. (2021) used bioprinted hydrogels containing metformin nanocarriers to promote the differentiation of human deciduous tooth stem cells into bone tissue while maintaining cell viability [119]. In addition, bioprinted hydrogels incorporating nanometal particles or bioactive molecules promote the differentiation of different oral stem cells into periodontal and bone tissue [120,121]. Furthermore, one study conducted in 2019, utilized a heparin-collagen gel containing BMP-2 supported by a bioprinted bioceramic scaffold. This combination induced the osteogenesis of dental pulp MSCs in vitro and resulted in ectopic bone formation in a rat model [14].

These studies collectively demonstrate the potential of cell-laden constructs fabricated by bioprinting for controlled drug release in dental tissue engineering applications. By incorporating drugs, growth factors, or bioactive molecules, these constructs can promote the tissue-specific differentiation of dental stem cells, leading to enhanced tissue regeneration and potential future clinical applications.

Another typical example of cell constructs with a bioactive factor was demonstrated in 2020 by in vitro studies of human DPSCs cultured in a BMP-2 incorporated GelMA/hyaluronic acid/glycerol bioconstruct [122]. As a result of the inclusion of BMP-2 growth factor, the expression of OCN and DSPP was significantly pronounced in human DPSCs. These bioactive factors can easily be blended with bioinks for bioprinting. In 2019, a decellularized dentin matrix that contains these bioactive factors was incorporated into an alginate hydrogel to enhance the odontogenic activities of cells (Figure 4d) [116].

A multi-tissue cell-laden structure for periodontal regeneration was studied in 2021 (Figure 4e) [117], and the construct was comprised of gingival fibroblast cell-laden collagen and strontium-doped calcium silicate. Using a bi-layered structure, in vitro experiments were used to assess the effects of the construct on cellular regeneration. They observed a significant increase in the secretion of fibroblast growth factor-2, BMP-2, and VEGF in human gingival fibroblasts. The construct stimulated the secretion of ALP, bone sialoprotein, and OCN. To evaluate the regenerative potential of the multicell-laden structure, animal models of osteoporosis demonstrated the enhanced regeneration of osteoporotic bone when the construct was utilized. These findings suggest that the multicell-laden structure effectively induced osteogenesis and guided periodontal regeneration, thereby highlighting the properties of a bi-layered cell-laden construct consisting of gingival fibroblast cell-laden collagen and strontium-doped calcium silicate to promote periodontal regeneration and facilitate efficient osteogenesis [117].

The applications of bioprinting using cell-laden bioinks in dental tissue engineering include cell construct fabrication, dentin regeneration, periodontal ligament regeneration, and drug (or growth factor) delivery systems. These applications highlight the versatility and potential of bioprinting technology to advance dental treatments, regenerative therapies, and personalized dental solutions.

## 6. Challenges and Future Perspectives

Developing new polymeric materials with suitable rheological properties, biocompatibility, bioactivity, and mechanical strength is a challenge in bioprinting. The complexity lies in ensuring that the biomaterial can successfully maintain structural integrity during printing and provide support for cell viability, proliferation, and differentiation, while maintaining structural integrity during printing [123]. To address this issue, an appropriate selection of polymeric biomaterials is required.

Achieving proper biomimetic tissue engineering in which printed constructs accurately mimic the complexity of natural dental tissues is challenging. The further development of the 3D bioprinting process is also necessary to improve resolution and printing technology. This includes a rapid printing process that enables the fabrication of complex cell-guiding micro/nanostructures and scaling-up of the technology to produce more biomimetic scaffolds [124]. Furthermore, by considering the complexity and adaptability of dental tissues to the native tissue environment, four-dimensional (4D) bioprinting, which integrates 3D printing with time, could be an alternative approach to enhancing the printing or complex shape design capability of current 3D bioprinting, considering the complexity and adaptability of dental tissues to the native tissue environment [125,126]. 

Stem cells pose another challenge in dental tissue engineering, as they are an inexhaustible source of cells for human organ regeneration. An optimally selected bioprinting process combined with undifferentiated cells (e.g., MSCs and *induced pluripotent stem cells*) could provide a technical opportunity to fully regenerate an entire tooth. Although hDPSCs are widely used in dental tissue engineering, adipose-derived stem cells are also a promising alternative source of abundant and available stem cells because they have the potential to differentiate into dental pulp, dentin, cementum, and periodontal ligaments [127,128].

Another important challenge is the lack of vascularization within bioprinted constructs for dental tissue engineering [129]. A proper blood supply is crucial for the survival, nutrition, and integration of regenerated tissues. Therefore, several studies have focused on developing strategies to promote the formation of functional blood vessels within bioprinted constructs. Prior research has provided empirical evidence that the inclusion of VEGF in bioconstructs containing DPSCs resulted in the modification of cellular phenotype through the stimulation of endothelial differentiation [130].

Advancements in multi-material bioprinting techniques will enable the fabrication of more complex dental constructs, including a whole tooth. For instance, in 2019, the combination of heparin, collagen, and BMP-2 was determined to meaningfully upregulate the odontogenic activities of DPSCs and formation of ectopic bone in rat model [14]. By combining multiple polymers, cells, growth factors, and other bioactive agents within a single-printed scaffold, it is possible to replicate the heterogeneity and functionality of natural dental tissues. Integrating bioprinting with other biofabrication technologies including electrospinning or 3D biofabrication methods, including microfluidic systems, may enable the creation of a more sophisticated dental tissue constructs. Complementary advantages can be achieved by combining different techniques to address specific challenges in dental tissue engineering.

Finally, the future of bioprinting in dental tissue engineering lies in personalized medicine. Periodontal tissues may exhibit diverse injury and damage geometries, depending on the patient. Thus, bioprinting technologies, combined with advanced imaging systems, can facilitate the generation of patient-specific dental constructs that match the individual anatomical and functional needs. Moreover, the effective tissue integration of the implanted 3D bioconstructs with the native periodontal tissue is a crucial factor to consider. The use of autologous tissues derived from patients is considered an important factor for the fabrication of dental constructs that are customized to individual anatomical and functional needs. This involves the formation of vascular and nerve networks in dental pulp tissues, as well as the anchoring of the teeth within the alveolar bone by cementum and periodontal ligament tissues [131].

## 7. Conclusions

Dental tissue regeneration holds significant promise in addressing the challenges associated with dental tissue defects, injuries, and diseases. In this review, the current advances in biomaterials and bioprinting applications in dental tissue engineering have been discussed. Through the integration of polymeric biomaterials and bioprinting, substantial progress has been made in the regeneration of dental tissues, dental bone, periodontal ligament, and dentin. However, there are several avenues available for future research and development, including the integration of functional 3D bioprinting with advanced imaging and autologous tissue implantation, to further advance this field and translate innovative strategies into clinical practice. This review provides a comprehensive overview of the current state of research on dental tissue regeneration, highlighting the importance of understanding the utilization of various approaches, including stem cells, scaffold materials, and bioprinting technologies.

## Figures and Tables

**Table 1:** Polymeric biomaterials for dental tissue regeneration (natural, synthetic, and hybrid polymer, and polymer-based composites).

Type	Polymeric Materials	Culturing Cells & Growth Factors	Dental Tissue	Outcomes	Limitations	Ref.
Natural polymers	Collagen	Erythropoietin. Vascular endothelial growth factor (VEGF).	Alveolar ridge augmentation	Enhanced alveolar bone regeneration. Enhanced vascularization.	Acellular implantation.	[62]
		Collagen sponge.	Periodontal ligament	Larger blood vessel area. Higher regenerated periodontal ligament compared to defect.	Insufficient tissue integration. Insufficient mechanical properties.	[77]
		Human periodontal ligament fibroblasts. Riboflavin.	Periodontal ligament	Enhanced periodontal tissue regeneration in rat model. Epithelial and connective tissue regeneration. Appropriate cellular alignment	Insufficient mechanical properties.	[63]
		Collagen sponge.	Alveolar bone	Increased cell attachment and alkaline phosphatase (ALP) activities compared to poly(glycolic acid) (PGA) scaffold. After 25 weeks implantation, complex dentin and cementum-like tissues formed.	Insufficient mechanical properties. Long regeneration duration.	[78]
		Polyphosphate (cross-linking agent). Bone marrow mesenchymal cells.	Alveolar bone	Enhanced mechanical strength. Improved bleeding control. Increased alveolar bone regeneration.	Toxic post-crosslinking required.	[79]
		Dental Pulp Stem Cells (DPSCs). Dentin Matrix Protein 1.	Dentin	Scaffold containing DPSCs, dentin matrix acidic phosphoprotein 1 (DMP1) and collagen significantly promotes dentin regeneration. Enhanced differentiation of DPSCs into odontoblast-like cells.	Insufficient mechanical properties. Lack comparison with Sham model to verify the findings.	[80]
	Alginate	TGF-β1.	Dental pulp	Upregulation of dentin matrix secretion. Induced differentiation of odontoblast-like cells. Enhanced natural regenerative capacity of dental pulp.	Complex three-dimensional (3D) structures cannot be formed. Insufficient mechanical properties. Difficulties in deployment of alginate/TGF-β1. Alginate polymeric matrix limits bioactivity.	[69]
		Laponite. DPSCs. VEGF.	Dental pulp	Injectable microspheres allowed controlled release of VEGF and laponite. Enhanced deposition of fibronectin and collagen type 1. Upregulation of new microvessel formation.	Lack of positive and negative controls to verify the findings. During injection process, large (~200 μm) microsphere may fragment due to low mechanical properties.	[70]
	Fibrin	DPSC-derived extracellular vesicles (DPSC-EC).	Dental pulp	Increased VEGF release. Enhanced vascularization of dental pulp tissue.	Insufficient in vivo data to verify in vitro findings. Extraction of DPSC-EC is costly with low yield.	[76]
	Hyaluronic acid (HA)	Odontoblastic cell line (KN-3 cells). Hyaluronic acid sponge.	Dental pulp	Lower granulated leukocytes compared to collagen scaffold suggesting lower immune response.	Gene expressions of IL-6 and TNF-α in KN-3 was similar in HA and collagen scaffolds.	[81]
		Human bone marrow mesenchymal stem cell (hMSCs).	Dental pulp	Enhanced osteo/odonogenic activities of hMSCs cultured in HA compared to mitrigel.	Lack of in vivo results to validate in vitro findings.	[82]
	Chitosan	TGF-β.	Dental pulp capping	Sustained release of TGF-β elevated cellular proliferation of odontoblast. 3~6-fold enhanced dentin formation compared to natural healing.	Concentration of TGF-β containing microsphere is not optimized. Limited angiogenic properties.	[83]
Synthetic polymer	polycaprolactone (PCL)	Nanohydroxyapatite. DPSCs.	Dentin tissue	Osteo/odontogenic activities (ALP and ARS expressions). Increased hydrophilicity. Enhanced cellular proliferation. Upregulation of BMP-2, RUNX2, and DSPP.	Difficulties in fabricating 3D geometries of dentin. Lack of in vivo results to validate in vitro findings.	[17]
		Mesenchymal stem cells. Platelet-rich plasma. β tricalcium phosphate.	Mandibular tissue	Enhanced bone regeneration around dental implants (increased bone-implant contact ratio and new bone height formation).	Insignificant difference in new bone area between groups.	[84]
	Poly(lactic acid) (PLA)	Human periapical cyst mesenchymal stem cells (hPCMSCs). Dicalcium phosphate dihydrate (DCPD). Hydraulic calcium silicate.	Periapical and alveolar bone	2.5-fold DMP-1 expression to pristine PLA scaffold. Adequate mechanical properties.	Acidic degradation of PLA. Lack of in vivo results to validate in vitro findings.	[85]
	Poly(lactic-co-glycolic acid) (PLGA)	Minocycline. Osteoblasts extracted from cranial bone of Newborn Sprague–Dawley rats.	alveolar bone	Improved hydrophilicity via electrospinning fabrication method. Promote attachment of osteoblasts.	Complex 3D geometries cannot be fabricated.	[15]
		--	Alveolar bone	Enhanced lamellar bone formation in alveolar tissue.	Insufficient comparative data (no positive and negative control). Complex 3D geometries cannot be fabricated. Acidic degradation of PLA.	[16]
Hybrid polymer	Chitosan/fibrin	hDPCS	Dental pulp	Incorporation of chitosan enhanced antibacterial effects without affecting cellular activities. Can provide endodontic space disinfection.	Lack of in vivo results to validate in vitro findings.	[86]
	Gelatin/fibrin	hDPCS	Dental pulp	Fibrin incorporation to gelatin enhances biomineralization. Enhanced biomineralization can increase mechanical properties. Upregulation of osteo/odontogenic related genes.	Lack of in vivo results to validate in vitro findings.	[87]
	Poly-γ-glutamic acid/glycerol/gellan gum	MG 63.	Alveolar bone	Careful selection of crosslinking ratio allowed strengthening of the polymeric matrix. Improved cellular proliferation and osteogenesis of MG 63.	In vitro cellular evaluation using MG 63 may not provide accurate results of guided alveolar bone regeneration. Lack of in vivo results to validate in vitro findings.	[88]
	Chitosan-collagen	Bone morphogenetic protein (BMP-7). Plasmid. hDPSCs.	Dental pulp	Controlled BMP-7 release in experimental group improved ALP activities and calcium deposition of hDPSCs. Upregulation of OCN, BSP, DSPP, and DMP-1.	Lack of in vivo results to validate in vitro findings. Complex microarchitectures cannot be formed. Insufficient positive and negative control comparison to determine the regenerative efficacy.	[89]
	chitosan–gelatin	Bone marrow mesenchymal stem cells.	Alveolar bone	Significant improvements in new bone formation. Increased neovascularization.	High inflammation reaction at early stages of implantation (day 5). Insufficient positive and negative control comparison to determine the regenerative efficacy. Complex microarchitectures cannot be formed.	[90]
		hDPCs.	Alveolar bone	Increased concentration of glutaraldehyde crosslinking agent prolonged the scaffold degradation. Enhanced osteo/odontogenesis-related genes (DSPP, BMP-2, IBSP, BGLAP, Osterix, and ALP). Improved mineralization.	Insignificant cellular proliferation rate compared to control. Subcutaneous in vivo results may not reflect the regeneration of alveolar bone. Complex microarchitectures cannot be formed.	[91]
	PCL/PEG	Rat BMSCs	Periodontium	Aligned electrospun PCL/poly(ethylene glycol) (PEG) fibers improved mechanical properties and provided contact guidance to cells. Upregulation of Col I, Col III, Postn, S100a4, ALP, and BGLAP gene expressions in aligned construct.	Unable to fabricate precise 3D construct. Requires complex post-fabrication procedures.	[92]

hPLFs (human periodontal ligament fibroblasts); bMSCs (bone marrow mesenchymal cells); DPSCs (dental pulp stem cells); PCMSCs (periapical cyst mesenchymal stem cells); VEGF (vascular endothelial growth factor); (*ALP*) (alkaline phosphatase); (*DMP*) (dentin matrix acidic phosphoprotein); *BMP* (bone morphogenetic protein); *IL6* (interleukin 6); *TNF* (tumor necrosis factor); *TGF* (transforming growth factor); *DSPP* (dentin sialophosphoprotein); *IBSP* (integrin binding sialoprotein); *OCN* (osteocalcin); *OPN* (osteopontin); *Runx* (runt-related transcription factor); BGLAP (bone gamma-carboxyglutamate protein); *Col* (collagen); *Postn* (periostin); *S100a4* (S100 calcium binding protein A4).

## Data Availability

Data are available from the corresponding author on request.
